# Untargeted Metabotyping *Lolium perenne* Reveals Population-Level Variation in Plant Flavonoids and Alkaloids

**DOI:** 10.3389/fpls.2017.00133

**Published:** 2017-02-07

**Authors:** Mingshu Cao, Karl Fraser, Chris Jones, Alan Stewart, Thomas Lyons, Marty Faville, Brent Barrett

**Affiliations:** ^1^AgResearch Grasslands Research CentrePalmerston North, New Zealand; ^2^PGG Wrightson SeedsChristchurch, New Zealand

**Keywords:** metabolomics, chemotyping, flavonoid glycosides, plant alkaloids, perennial ryegrass

## Abstract

Metabolomics provides a powerful platform to characterize plants at the biochemical level, allowing a search for underlying genes and associations with higher level complex traits such as yield and nutritional value. Efficient and reliable methods to characterize metabolic variation in economically important species are considered of high value to the evaluation and prioritization of germplasm and breeding lines. In this investigation, a large-scale metabolomic survey was performed on a collection of diverse perennial ryegrass (*Lolium perenne* L.) plants. A total of 2,708 data files, derived from liquid chromatography coupled to high resolution mass spectrometry (LCMS), were selected to assess the effectiveness and efficiency of applying high throughput metabolomics to survey chemical diversity in plant populations. The data set was generated from 23 ryegrass populations, with 3–25 genotypes per population, and five clonal replicates per genotype. We demonstrate an integrated approach to rapidly mine and analyze metabolic variation from this large, multi-batch LCMS data set. After performing quality control, statistical data mining and peak annotation, a wide range of variation for flavonoid glycosides and plant alkaloids was discovered among the populations. Structural variation of flavonoids occurs both in aglycone structures and acetylated/malonylated/feruloylated sugar moieties. The discovery of comprehensive metabolic variation among the plant populations offers opportunities to probe into the genetic basis of the variation, and provides a valuable resource to gain insight into biochemical functions and to relate metabolic variation with higher level traits in the species.

## Introduction

Perennial ryegrass (*Lolium perenne* L.) is a widespread species in pasture and amenity settings throughout temperate latitudes. Compared to other forage species such as *Festuca* spp., the value of perennial ryegrass generally lies in its rapid establishment, long growing season, high palatability, and digestibility for ruminant animals (Humphreys et al., 2010). To date the main focus of ryegrass improvement has been on yield, heading date, disease resistance, seasonal growth, and persistence (Williams et al., 2007). Perennial ryegrass is an obligate outcrossing species with limited information on genetic regulation of the targeted (often complex) traits. This knowledge limitation may be part of the reason why the rate of genetic gain for forage yield achieved in this species has been modest (Brummer and Casler, 2014).

Some of the most important aspects of dissecting a complex trait is to accurately characterize the target trait and trait components, and to elucidate genetic and environmental factors affecting the trait. For example, enhanced grass digestibility in ruminants is of long standing research interest. Metabolites associated with aspects of digestibility have been reported including phenolic acids (Theodorou et al., 1987), lignin (Vogel and Jung, 2001), and water soluble carbohydrates (Rasmussen et al., 2012). However, the underlying metabolic mechanism for digestibility of grasses is far from clear. Global metabotyping, i.e., phenotyping at the metabolic level, may offer new opportunities to identify all the chemical components, followed by modeling their combined effects on digestibility or other complex traits. High-throughput metabolomics not only provides the power to survey natural metabolic variation of known and novel molecules among and within populations, it also enables accurate detection of associated genes (Dumas et al., 2007). Across a range of species, metabolomics has become a powerful platform which can be used to investigate genetic diversity at the metabolic level (Fernie and Klee, 2011; Langridge and Fleury, 2011), to draw associations between metabolic phenotypes and morphological traits (Hu et al., 2014), and to identify genetic loci (Koulman et al., 2009) or to clone genes governing the accumulation of biologically important metabolites (Dumas et al., 2007).

Due to the dynamics and structural complexity of natural metabolites different analytical platforms have to be employed to investigate each class of metabolites. Thus, the types of metabolites that can be measured are necessarily limited by different analytical methods in extraction, separation and detection. In this study, a non-targeted metabolomics platform using liquid chromatography (reversed phase (RP), hydrophobicity based) coupled to high resolution electrospray ionization (ESI) mass spectrometry (LCMS) was employed (Fraser et al., 2014), to enable ready detection of semi-polar compounds.

In perennial ryegrass, metabolic research interests have focused on plant-endophyte interactions (Cao et al., 2008; Rasmussen et al., 2008; Ponce et al., 2009; Qawasmeh et al., 2012), with little effort pursued on the natural metabolic variation of the host plant itself. The objectives of this study were: (1) to evaluate the reproducibility of LCMS-based metabolomics from large, multi-batch experiments; (2) to study metabolic profiles within and among ryegrass populations and; (3) to discuss the opportunities and limitations of high throughput metabolomics in genetic studies and improvement of ryegrass, and outcrossing plant species in general.

## Results

### Data Quality Evaluation of the Detected Peaks

Thirty-two thousand, two hundred and seventy-three peaks from positive ESI and 20,838 peaks from negative ESI were detected from 1,331 to 1,377 samples, including controls, respectively. For the convenience of discussion, a nomenclature for peaks is assigned as for example, CP434.2167_4.58, where CP represents the C18 column in positive ion mode, 434.2167 the measured *m/z* and 4.58 the retention time in minutes. Accordingly, CN denotes peaks from the negative ESI.

**FIGURE 1:**
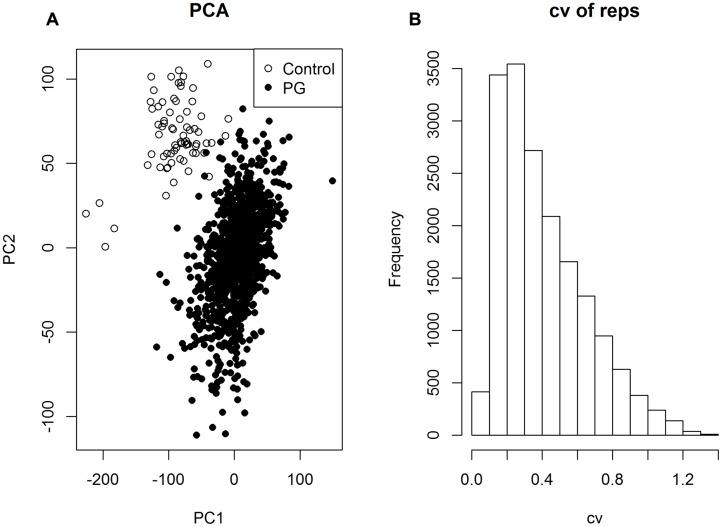
**(A)** Principal component analysis analysis based on 28,043 CP peaks from 1,162 experimental samples (PG group) and 71 controls (Control group). **(B)** Distribution of the coefficient of variation (cv) of all the peaks, evaluated based on the replicates (*n* = 5) of each genotype.

After applying a series of quality control filtering, including peak merging, batch effect normalization, and de-isotoping procedures 28,043 CP peaks were retained. Overall quality was evaluated by principal component analysis (PCA) where control samples (*n* = 71) are differentiated from all experimental samples (the PG group, *n* = 1162) (**Figure 1A**) in a space spanned by PC1 (5.2%) and PC2 (4.1%). The separation of control samples demonstrates the effectiveness of the peak filtering procedures for correcting the batch effect which was the predominant variation prior to filtering (**Supplementary Figure 1**). Similar results were found for 15,486 CN peaks (**Supplementary Figure 2**).

Excluding the control samples, a statistical filtering (Kruskal test, *p*-values < 0.05) was applied to retain peaks of biological interest among the 23 populations. This led to 17,579 CP peaks from 1,162 PG samples. To assess the technical variability and evaluate the quality of individual peaks, we first calculated the coefficient of variation (cv) of the five replicates in each of 233 genotypes, and the median value of cv among all genotypes. Among all 17,579 CP peaks, 42.1% of peaks exhibited cv < 0.3 (**Figure 1B**), indicating appreciable variation but an adequate number of peaks measured with certainty to justify peak annotation.

**FIGURE 2:**
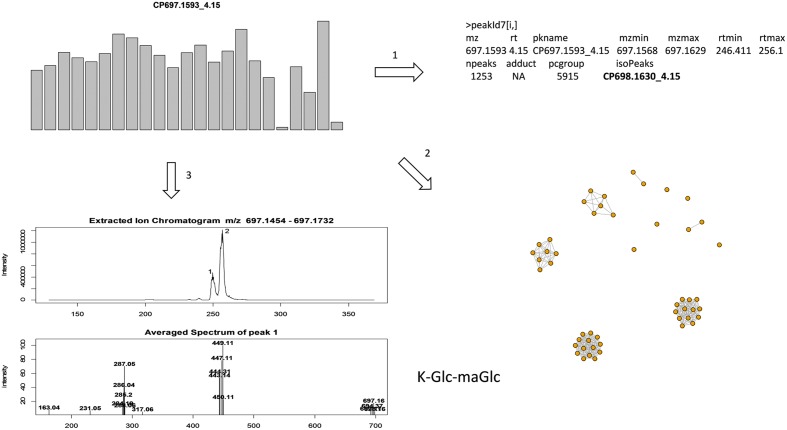
**Illustration of annotation methods.** To annotate a targeted peak CP697.1593_4.15, it’s isotopic peak (CP698.1630_4.15) must be present in the peak annotation table, and the peak retention time must be located in [rtmin, rtmax] in the peak annotation table (step 1); use correlation structure of top ranked peaks so that highly correlated peaks (*r* > 0.9) can be annotated together, with co-eluting peaks often assigned as fragment ions (step 2); extracted ion chromatogram (EIC) of *m/z* 697.1593 ± 20 ppm was retrieved from a sample with strong peak intensity, the average mass spectrum was constructed from each individual peak (step 3). Here the mass spectrum of peak 1 at 4.15 (249 s), with co-eluting ions *m/z* 287.05, 449.11, can be annotated as K-Glc-maGlc. Peak 2 is an isomeric peak of CP697.1593_4.15, with characteristic ions *m/z* 287.05, 449.11 and 535.11. The presence of *m/z* 535.11 (–86) further suggests the presence of a malonyl group in the molecule.

Data quality should comprise both the reproducibility of peak intensity and the reliability of peak identity. However, in large scale metabolomics studies peak complexity often confounds peak identification due to a mixture of compounds within a peak or the difficulty in peak alignment. In this study, as illustrated in **Figure 2**, we follow three criteria for annotation: (1) isotopic peak presence, e.g., to annotate the peak of interest CP697.1593_4.15, its isotopic peak (CP698.1630_4.15) must be present in the peak annotation table; (2) use of the correlation structure of top ranked peaks to enable a group of peaks, often co-eluting, to be annotated simultaneously rather than on an individual basis; and (3) search in the raw data generated from highly expressed samples and compute the mean mass spectrum of the eluting peak for spectral interpretation. Any peaks deficient in these aspects of information are largely excluded from discussion in this study. As an example (**Figure 2**), the extracted ion chromatogram (EIC) of *m/z* 697.1593 ± 20 ppm was retrieved from a sample with a strong peak intensity and the mean mass spectrum was constructed from individual peaks. The mass spectrum of peak 1 at 4.15 min (249 s), with co-eluting ions *m/z* 287.05, 449.11 indicates the presence of aglycone kaempferol (K) and the release of hexose (*m/z* 162) moieties; the loss of *m/z* 248.05 indicates the presence of a malonyl hexose (maHex). We assume *m/z* 162 as glucose (Glc) as it is the most commonly found hexose in plant flavonoid *O*-glycosides. Therefore, CP697.1593_4.15 can be annotated as K-Glc-maGlc, where maGlc represents maHex. A loss of *m/z* 146 can be attributed to rhamnose (Rha) because it is the only deoxyhexose known to form natural flavonoid conjugates.

### Distinct Metabolic Profiles in Populations of North African Origin

The median peak intensity among the five clonal replicates was used to assess clonal variation among the 233 genotypes (individual plants). Those peaks that are invariant (Kruskal test, *p*-value > 0.05) among the 23 populations (including cultivars, ecotypes and breeding lines) were removed. 17,579 CP peaks and 12,238 CN peaks were consequently retained to investigate the pattern of variation among the populations.

**FIGURE 3:**
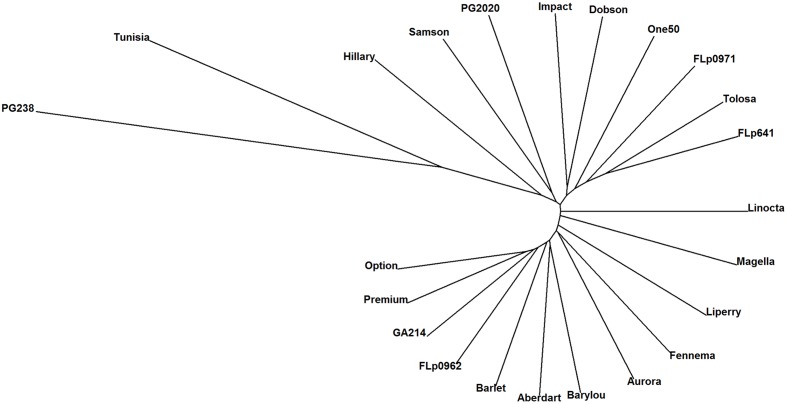
**UPGMA clustering of 23 ryegrass populations (Euclidean distance metric was used; based on 17, 579 CP peaks).** PG238 and Tunisia populations show profiles distinct from all others.

Based on the 17,579 CP peaks the UPGMA (Unweighted Pair Group Method with Arithmetic Mean) clustering of 23 ryegrass populations reveals ‘PG238’ and ‘Tunisia’ are clearly differentiated from the others (**Figure 3**). Both populations are of North African origin, ‘PG238’ being a Moroccan ecotype and ‘Tunisia’ a Tunisian ecotype. All other populations are European and New Zealand cultivars or breeding lines.

An empirical Bayes method (Smyth, 2004) was used to identify peaks differentiated among the populations. Many CP/CN peaks were shown to be significantly different, with FDR (false discovery rate) adjusted *p*-value < 0.01, among the 23 populations. With a test on the 711 most intense CP peaks (with mean intensity > 1e5), 551 (77.5%) were found to be significant and likewise, 86.5% (365/422) of the CN peaks were significantly different. Here, we only focus on the intense peaks (which likely give rise to more informative spectra), and use a ranking algorithm (Ahdesmäki and Strimmer, 2010) to select the top 50 most differentiated peaks to carry out annotation.

**FIGURE 4:**
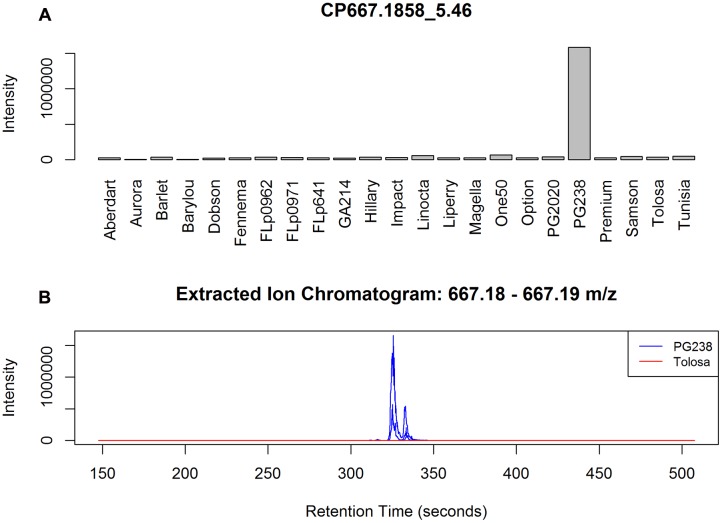
**(A)** Example of a PG238 specific peak CP667.1858_5.46, annotated as I-acGlc-Rha. The barplot is based on the median averaged peak intensity of all the samples from the populations. **(B)** EIC plots of three randomly selected data files from PG238 and Tolosa, respectively. Chromatographic peaks, although shifting, are well formed in PG238 samples but no signals detected from Tolosa samples.

Among the top ranked CP/CN peaks (**Supplementary Data 1** and **Data 2**) many are only present or absent in ‘PG238.’ An example is peak CP667.1858_5.46 (**Figure 4**), which can be annotated as isorhamnetin-acetylglucosyl-rhamnoside. Our annotation reveals that the top ranked CP/CN peaks represent a class of flavonoids and their derivatives, which can be characterized by typical fragment ions in mass spectra. Briefly, the backbone of this class of molecules can be identified by the characteristic accurate *m/z* in positive ion mode, with *m/z* 287.05 of kaempferol (K), *m/z* 303.05 of quercetin (Q) and *m/z* 317.06 of isorhamnetin (I), in protonated form [M+H]^+^, respectively. These three main flavonols are commonly found in *Lolium* spp., often existing as *O*-glycosides (Qawasmeh et al., 2012). Sugar conjugates are also found acetylated with a typical loss of *m/z* 204, and acetylhexose is denoted as acGlc, hereafter. As a result, a number of annotated metabolites, such as I-acGlc-Rha (CP667.1858_5.46, CN665.2085_5.39), I-acGlc (CP521.1286_5.89), Q-acGlc-Rha (CP653.1703_5.11) and Q-acGlc (CP507.1128_5.59, CN505.0985_5.54), are only present in ‘PG238.’ Among the top ranked peaks (**Supplementary Data 1** and **Data 2**) are also fragment peaks, which are correlated and co-eluting, for example CN300.0272_5.55 is a fragment peak from CN505.0985_5.54.

Another group of metabolites that are absent in both ‘PG238’ and ‘Tunisia’ include K-Glc-maGlc (CP697.1593_4.15, CP535.1073_4.15, see it’s annotation in **Figure 2**), and K-Glc (CP449.1071_4.04, CP287.0545_4.04). K-Glc-maGlc can also be confirmed by its presence in the top CN peaks as CN695.133_4.13. Notably, among three isomeric peaks both CN651.1567_5.07 and CN651.1564_5.29 are present only in ‘PG238,’ whereas CN651.1563_4.13 is absent. Interrogation of the raw data indicated the two peaks present in ‘PG238’ are Q-derivatives; and the absent peak is the K-derivative (K-Glc-maGlc).

Further to this, CP288.1590_5.17 is present only in ‘Tunisia.’ We predicted its formula as C_17_H_22_NO_3_ ([M+H]^+^) and annotated the peak as thesinine. This peak formed its own group in peak clustering analysis (**Supplementary Data 2**). However, thesinine rhamnoside (The-Rha), detected as CP434.2167_4.80, among the top ranked peaks (**Supplementary Data 1**, to be discussed further in the following section), is only present at a very low level in ‘Tunisia.’

To summarize, ryegrass populations can be effectively separated by mass signals without metabolite identification. Subsequent peak annotations revealed structural differences in flavonoids amongst the ryegrass populations, where flavonoid glycosides uniquely present in ‘PG238’ are all Q (Q-acGlc-Rha, Q-acGlc) and I derived (I-acGlc-Rha, I-acGlc), but not K derived. K derived flavonoid glycosides including K-Glc-maGlc and K-Glc were found absent in both ‘PG238’ and ‘Tunisia.’ The unique presence of thesinine in the ‘Tunisian’ population indicates its specific regulation of thesinine, whose relation to The-Rha remained to be explored.

### Metabolic Variation among Populations Excluding North African Origin

Populations ‘PG238’ and ‘Tunisia’ have very different metabolic features from the other 21 populations, evident by the qualitative variation amongst the top 50 ranked CP/CN peaks. To further assess quantitative differences in metabolic profile among the rest of the perennial ryegrass populations, we exclude the two populations from the following analysis.

**FIGURE 5:**
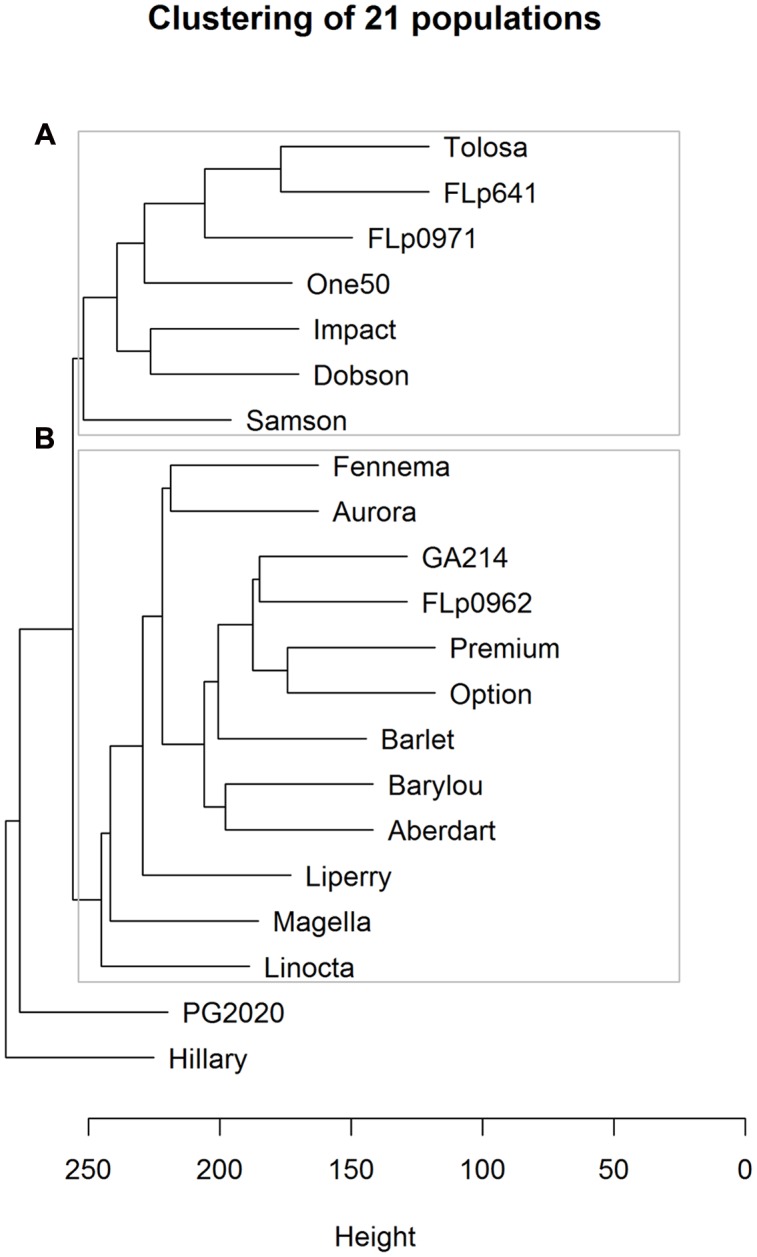
**UPGMA clustering of 21 populations using Euclidean distance metric among 17,579 CP peaks.** Cluster A represents the populations of New Zealand (NZ) origin and cluster B of European origin.

Linear discriminate analysis was initially applied to all of the 1,057 samples (genotypes × reps) with 17,579 CP peaks partly revealing population structures (**Supplementary Figure 3**). While discriminate analysis may overfit as it aims to maximize separation between groups, all the genotypes from a given population formed a single cluster suggesting good data quality overall. Using UPGMA clustering analysis we investigated relationships among the 21 populations, revealing that the metabolic profile is strongly associated with population provenance (**Figure 5**). Largely, two sub-clusters are formed comprised of populations in cluster A of New Zealand (NZ) origin and in cluster B of European origin. More specifically, both the populations ‘Option’ and ‘Premium’ were cultivars bred in Northern Europe; and ‘GA214’ and ‘FLp0962’ were developed from similar European accessions. Within cluster A there is some differentiation between ‘Samson,’ a cultivar bred from NZ-adapted material, and cultivars and breeding lines wholly- or partly derived from germplasm originating in the North West of Spain. ‘PG2020’ originates from crosses between ‘PG238’ and breeding lines containing NZ and North West Spain germplasm. ‘Hilary’ is a selection from the older NZ cultivar ‘Ruanui.’ Encouraged by the distinct patterns that are associated with breeding history we then aimed to identify metabolites specific to populations.

**FIGURE 6:**
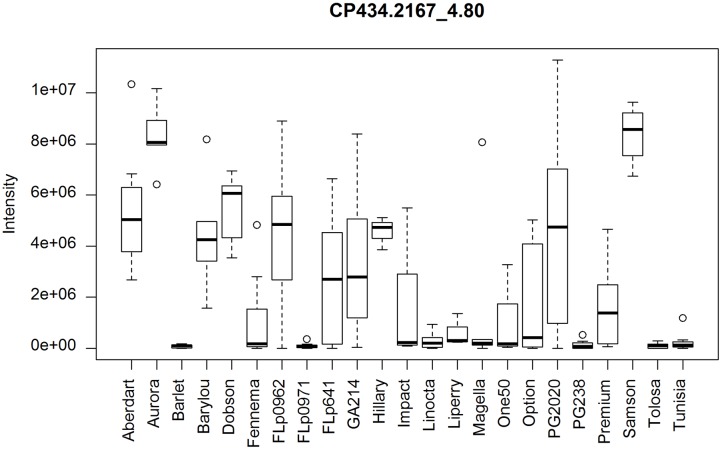
**Distribution of thesinine rhamnoside (CP434.2167_4.80) abundance among the populations.** PG238 and Tunisia lack this compound. Wider variation occurs within the FLp0962, PG2020, GA214, and FLp641 population samples.

Using the same ranking algorithm described we identified the top 50 differential CP and CN peaks among these 21 populations. The most varied metabolite, as shown on the top ranked list (**Supplementary Data 3**) is The-Rha (**Figure 6**). The-Rha is represented by the peak CP434.2167_4.80 and its fragment peak CP288.159_4.80. Another isomer of The-Rha, CP434.2167_4.58, highly correlated with CP434.2167_4.80 (*r* > 0.98, *n* = 23), is also present in the top list. Two isomers of E/Z-thesinine-O-4’-alpha-rhamnoside have been structurally elucidated in ryegrass (Koulman et al., 2008). By using direct infusion mass spectrometry (DIMS) The-Rha showed significant genetic variation between cultivars ‘Samson’ and ‘Impact,’ being more abundant in ‘Samson’ (Koulman et al., 2009), which is also seen here (**Figure 6**).

Among the top varied metabolites is K-Glc-maGlc, represented by the top peaks CP697.1598_4.04, CP535.1075_4.04, CP449.1071_4.04, and CP287.0545_4.04. K-Glc-maGlc, which is absent from ‘PG238’ and ‘Tunisia’ as shown in the previous section, is present at a low level in ‘GA214,’ and at the highest level in ‘Tolosa.’ The annotation of this CP peak list indicates that K and its derivatives are notably varied among the 21 populations.

Annotating the two sets of top ranked peaks (**Supplementary Data 3** and **Data 4**) demonstrates the challenges in assigning peaks to metabolites. One challenge is that a peak may represent a mixture of compounds due to insufficient chromatographic separation. Peaks in this category include CP859.1917_5.21 (a mixture Q- and K-derivatives), CN695.1332_4.03, CN695.1468_4.24 [a mixture of chlorogenic acid (CGAs) derivative and K-derivative]. Other challenges are due to the limited in-source fragmentation for annotation, for example CP659.3629_8.94 and CP261.2209_8.94 and the peak mis-alignment, see discussion as follows.

**FIGURE 7:**
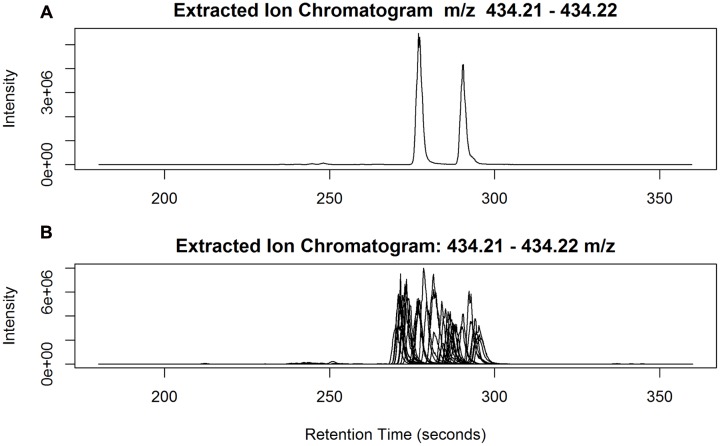
**(A)** Two isomeric peaks of The-Rha eluted at 4.63 min (278 s) and 4.83 min (290 s) in one sample; **(B)** The two peaks superimposed from all the 20 Samson samples, run in 18 different batches.

While it is challenging to establish systematic approaches to evaluate the reliability of peak identification among a large number of samples, our prior knowledge of the chemical structures of The-Rha in ryegrass (Koulman et al., 2008) offers a unique opportunity to examine this empirically. With its theoretical mass of 434.2173 [M+H]^+^ and a 20 ppm window, we found six peaks detected and captured in the peak annotation table. These six peaks have only *m/z* < 1.4 ppm error from the theoretical mass, but with much wider variation in peak retention, from 3.88 to 4.80 min. Although only two peaks can be discerned in each individual sample (**Figure 7A**), when all 20 samples from ‘Samson,’ run in 18 different batches, are taken together it becomes impossible to differentiate the retention time of the two peaks across batches (**Figure 7B**). As a result, six peaks were assigned in peak detection instead of two. Chromatographic peak mis-alignment may be unavoidable for closely eluted peaks in multi-batch experiments. Different peak alignment algorithms may be employed to evaluate their performance but can be time-consuming for large data sets (see Discussion). We undertook peak aggregation analysis by summing up all the detected isomeric peaks when the identification of isobaric metabolites among samples is evaluated as uncertain.

**FIGURE 8:**
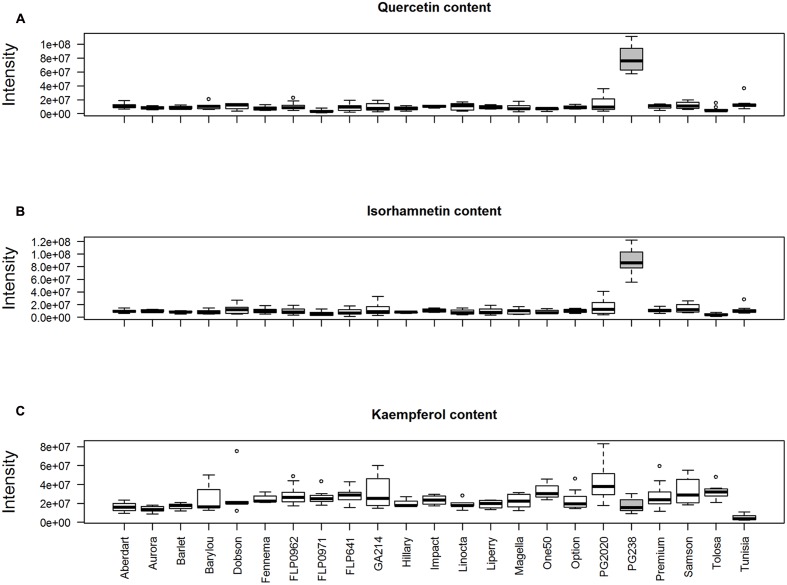
**Peak aggregation analysis of** **(A)** quercetin ([M+H]^+^, 303.0499 *m/z*, 15 peaks); **(B)** isorhamnetin ([M+H]^+^, 317.0656 *m/z*, 15 peaks) and **(C)** kaempferol ([M+H]*^+^*, 287.0550 *m/z*, 23 peaks).

We performed a peak aggregation analysis of quercetin (Q), isorhamnetin (I), and kaempferol (K) (**Figure 8**). This confirmed the observation based on individual peak analysis, that ‘PG238’ samples had high levels of Q and I. **Figure 8C** also confirms the extensive variation of K compared to Q and I among the populations (*F*-test, *p*-value < 2.2e-12), with ‘Tunisia’ having the lowest level of K and ‘PG2020’ having the largest within-population variation. However, it was noted that K, exhibiting complex chromatographic behavior, was not differentiated from its isomeric flavone luteolin using this coarse-level quantification.

Peak aggregation analysis was also performed for CGAs, which are represented by peaks CN353.0873_3.84, CN353.0874_4.06, CN353.0873_4.20, and CN353.0875_5.39 and their derivatives (**Supplementary Data 4**). CGAs are abundant in perennial ryegrass which has three isomers (Qawasmeh et al., 2012). Under this experimental condition three well-formed peaks for CGAs (353.0867, calculated *m/z*, [M-H]^-^) can be seen among samples. However, in total 55 peaks of 353.0867 (±10 ppm) were detected here due to misalignment from 3.5 to 4.5 min across samples. Peak aggregation analysis of CGA (CN353.0875) demonstrates that there is no difference among the 21 populations (*F*-test, *p*-value = 0.93). Of the four peaks among the top list only CN353.0875_5.39 shows a distinct profile, with the highest level present in ‘PG2020’ and lower or at the baseline noise level among other populations. The EIC data interrogation showed this peak can be sufficiently separated from the other three peaks. CN353.0875_5.39 is a relatively weak peak but consistently present in ‘PG2020’ genotypes (**Supplementary Figure 4**). CN353.0875_5.39, co-eluting with 515.12, could be annotated as CGA glycoside due to the neutral loss of *m/z* 162.

Perloline, one of the known alkaloids present in the *Lolium* and *Festuca* species, was detected as CP333.1174_5.28 (FDR adjusted *p*-value < 0.01), with 18 ppm error from the calculated *m/z* 333.1234, [M+H]^+^, and present as a single chromatographic peak in individual samples. CP333.1174_5.28 is not on the top 50 significant peaks (**Supplementary Data 3**) but among the list of significant peaks based on empirical Bayes, suggesting more informative metabolic variation can be mined from the data.

To summarize, plant alkaloids and kaempferol derived flavonoids are notably varied among the 21 populations. The population-level variation of The-Rha and perloline revealed here expands our knowledge on the magnitude of their variation in ryegrass. A CGA glycoside was found to be unique to ‘PG2020’ but CGAs were invariant among the populations. Structural variation of sugar moieties in flavonoids may contribute to ryegrass population differentiation. As examples, K-Glc-maGlc is most abundant in ‘Tolosa’ and K-Glc-feruloylGlc (**Supplementary Data 5**) is most abundant in ‘Samson,’ but both compounds are present at various levels, even down to noise level, in other populations.

As a further note, to assess whether these population-level variations are consistent across seasons and under various environments we have conducted peak validation with samples harvested in a different year (**Supplementary Data 6**). The results demonstrated two metabolites (K-Glc-maGlc and CGA glycoside) identified and annotated in this study were reproducible between two environments indicating the robustness of our statistical data mining procedures. The magnitude of population-level variation documented here serves as a resource to research how these metabolic variations are associated with high level traits or modulated by genetic regulation..

## Discussion

High-throughput metabolomics was demonstrated here as an effective tool in screening a diverse collection of plant germplasm for metabolic variation. However, to make such methods a routine practice in understanding complex traits at the metabolic level, and in searching for genes responsible for the metabolic variation, it is important to acknowledge both the opportunities and limitations of these methods.

### Addressing Peak Annotation Problems to Facilitate Biological Interpretation

Many thousands of peaks can be generated from high-throughput LCMS platforms. Currently, it is the common practice to use statistical testing and/or machine learning ranking algorithms to identify a smaller set of peaks for closer inspection of their chemical identities. This practice falls short of the demand to understand metabolism underlying trait expression in plants. When a large number of statistically significant peaks are identified, peak annotation remains a bottleneck in metabolomics studies (Wishart, 2011; Cao et al., 2013, 2015). Detailed mining and evaluation of chemical features from data, such as isotopic patterns, adduct ions, in-source fragmentation patterns, and co-eluting behavior must be undertaken for peak annotation, and it is still challenging to automate such complex annotation processes. Database searching is often used for annotation but, in our experience, this is more effective for modular molecules such as lipids (Cao et al., 2013). For small metabolites we have to collect more annotation features including tandem mass spectra (MS^2^) of targeted ions or predicted retention time, to arrive at MSI (Metabolomics Standard Initiative) level 2 identification (Sumner et al., 2007). An unexpected list of metabolites under a particular biological context is usually found, indicating that the extent of our picture of cellular metabolism is incomplete (Patti et al., 2012). The long list of unknown peaks of biological relevance strongly suggest more effort needs to be invested into “peakomics” before the full value of metabolomics can be realized. Peakomics should aim to understand the massive number of peaks generated from LCMS data, to devise a set of algorithms to integrate chemical features that can be mined from the data, and to draw associations of the mined chemical features with known metabolites. In this study, along with the data processing pipelines we maintained a peak annotation table for chemical features, and built a spectral database in text format to enable quick evaluation of peak identity in future studies. Although we handle isotopic peaks effectively, we need to improve our grouping algorithms to handle co-eluting peaks and to tackle peak deconvolution in high throughput LCMS.

Batch effect is perhaps the largest source of variation in metabolomics and analytical chemistry. Much research has been done on normalizing such systematic biases, but the aim has been exclusively to normalize peak intensity. As we have shown here mis-alignment across a large number of batches can lead to enormous uncertainty in peak identity. Peak identification and quantification are two sides of the same coin – wrong assignment of peaks can lead to a spurious list of significant peaks. Although we have approached the use of the predicted retention time for peak annotation (Cao et al., 2015), its reliable use in peak annotation can still be challenging in multi-batch LCMS experiments (Abate-Pella et al., 2015). There are no universal algorithms that can address all the problems. We have proposed and used peak aggregation analysis as a compromise approach. Different platform offers different level of resolution in metabolite identification. Once again taking The-Rha as an example, it is routinely identified by DIMS as nominal or accurate mass, by HILLIC-MS as a single peak (Cao et al., 2015) and by C18 LCMS as two isobaric metabolites. As for the metabotyping purpose further studies need to be conducted to evaluate how quantification can be compared across platforms and what level of details are required.

When quantification of isobaric metabolites is of a critical task in metabotyping dedicated algorithms should be developed to properly group isomeric peaks across batches based on EIC data. Some directions for this development may include using prior knowledge to group peaks if the number of isomeric peaks is known, as in the case for The-Rha. In addition, if MS^2^ data are available to support two peaks with wide retention time variation being the same metabolite (Cao et al., 2013), such information can be exploited to improve peak alignment. Finally, statistical modeling of EIC data may suggest the number of isomeric peaks that can be assigned with confidence. Only with accurate identification the quantification of isobaric metabolites can be ensured, then research into their biological regulation can be proceeded.

### Metabotyping Offers New Opportunities to Characterize Complex Traits

Phenotypic evaluation of the collected germplasm is a prerequisite for germplasm prioritization or breeding program design but it is not straightforward and often indirect. Metabotyping, dissecting a phenotype at the metabolic level, potentially enables more precise evaluation of forage quality traits than those of traditionally used assessments such as fiber content, metabolizable energy, etc. On the other hand, metabolites themselves may form the end phenotype which could be directly selected for. For example, the presence of defense compounds may be used to select for disease resistance.

The diversity of alkaloids and flavonoids is well understood in plants (Harborne, 1977). Alkaloids are believed to function in plant defense (Ziegler and Facchini, 2008) and flavonoids play a role in plant adaptation to the environment (Mouradov and Spangenberg, 2014). Our results showed that flavonoids and their derivatives are diverse among ryegrass populations and seem to be adaptive to local environments, which suggests this class of compounds is potentially responsive to selection. Greater understanding of the genetic regulation of this class of compounds in ryegrass will be our next step. Fortunately, we can learn from model species where the genetic control of flavonoids has been well documented, especially with the aid of metabolomics and genomics platforms (Saito et al., 2013). It becomes clear that genes are involved not only in the formation of aglycone structures but also their subsequent modifications. Extensive flavonoid variation was uncovered in the ryegrass populations, and the variation lies not only in the type of aglycones but also in sugar modifications. This provides information that will enable us to explore new frontiers in developing our understanding of the genetic basis of their regulation in ryegrass. First, we will need to draw associations between this class of compounds and higher level traits, e.g., in maize, the *C*-glycosyl flavones maysin and apimaysin are involved in corn earworm resistance (McMullen et al., 1998). Second, we can design experiments to research the genetic basis (of qualitative or quantitative nature) of a set of flavonoids and alkaloids in ryegrass that were revealed in this study. Third, we can specifically look into the function of acetylated/malonylated/feruloylated flavonols in ryegrass, as these decorated flavonoid glucosides have been shown to facilitate the transportation and accumulation of flavonoids in cellular compartments (Zhao et al., 2011).

The-Rha, as a metabolic trait, is subjected to genetic control in *L. perenne* with a strong QTL (quantitative trait locus) located in the linkage group 4 (Koulman et al., 2009). Although structurally elucidated its relation to high level traits such as pasture persistence remains unknown. Perloline also exhibits broad variation among the 23 populations (**Supplementary Figure 5**), and in contrast, functions of this alkaloid were reported in literature Intraspecific differences were observed earlier by Butler (1962); and a few major genes were reported to contribute to perloline heritability (Buckner et al., 1973). Because of its inhibitory effect on ruminal digestion developing low perloline forage has long been a breeding goal (Cornelius et al., 1974). Metabotyping a wide range of germplasm, therefore, may offer new opportunities to identify materials for developing low-perloline populations.

Phenotypic characterization often remains simplistic and the main bottleneck to understanding the genetic basis of traits (Myles et al., 2009). Metabotyping was demonstrated as an effective tool for us to understand metabolic variation within and between ryegrass populations, and may serve as a bridge to understanding the manifestation of economically important traits (Cao et al., 2012). A combined use of high throughput genotyping and metabotyping should provide a new avenue for us to discover novel associations between allele frequency and metabolic variation in ryegrass populations. Such integration will enable us to conduct targeted molecular breeding of the metabolites that contribute to the adaptive or nutritive value of forage species.

## Materials and Methods

### Plant Materials

Twenty-three populations were selected from a large collection of breeding lines, cultivars and wild ecotypes, which was designed for genetic association mapping. In this paper we refer to cultivars, breeding lines or ecotypes as populations, and each individual plants from population as genotypes due to the nature of high heterozygosity in perennial ryegrass. These 23 populations were selected to ensure that more than three genotypes were included from each population and five clonal replicates of each genotypes were available for population-level analysis. In total 233 genotypes from these populations were selected for this investigation (**Supplementary Table 1**). Seeds of all ryegrass material were treated by heat and fungicide to ensure that they were endophyte-free. All plants were grown in pots and placed outdoors at AgResearch Grasslands Research Centre, Palmerston North, New Zealand.

### Metabolomic Assay and Data Analysis Pipeline

Plants were harvested after 3 weeks regrowth (to mimic rotation grazing) by cutting all the tissues 4 cm above the pot surface, predominantly consisting of the leaf blades. Samples in this study were harvested in May 2012, late autumn in New Zealand. Control samples were derived from a bulk sample consisting of a collection of ryegrass materials that may contain endophyte. These control samples were formed to monitor systematic variations such as run order effect and batch effect. Sample preparation and Metabolomics lab analyses followed protocols previously reported (Fraser et al., 2014). Briefly, ground plant material (50 mg) was extracted in 50:50 acetonitrile-water (v/v), and analyzed by RP LC-MS via a Thermo Exactive mass spectrometer (Q-Exactive MS, Thermo, Waltham, MA, USA) with ESI. RP LC–MS analysis was performed using an Agilent RRHD SB-C18 column (150 mm × 2.1 mm, 1.8 μm) with a gradient elution of water containing 0.1% formic acid and acetonitrile containing 0.1% formic acid at a 400 μl/min flow rate. Samples were run in both positive and negative ionization mode as separate chromatographic runs. MS data were collected in profile mode over the mass range of *m/z* 60–1200.

The large number of biological samples had to be assayed in multiple batches. The biological samples (**Supplementary Table 1**) selected for this investigation were run in 36 randomized batches over 9 months. Data files generated from all the genotypes discussed above, and control samples from each batch, were subjected to data analysis. In total, 1,260 data files (plus 71 controls) in positive ionization mode and 1,297 data files (plus 80 controls) in negative ionization mode, were analyzed.

The profile (positive and negative ionization) mode data were converted into centroid mode using ProteoWizard tools (Holman et al., 2014). Peak detection, alignment, grouping and missing value filling procedures were conducted using the XCMS package (Smith et al., 2006) with key parameter settings as follows: peak detection was carried out on the centroid data using the “centWave” algorithm with peakwidth = 3:15 and ppm = 20; peak alignment was undertaken using the “obiwarp” method with default parameters and; peaks were grouped using the density-based approach. The resulting peak intensity tables were de-isotoped by CAMERA (Kuhl et al., 2012) and using our in-house peak de-isotoping procedures. Our de-isotoping of peak groups ([M], [M + 1], [M + 2]) was implemented as follows: the ion series of the peak group must be within ±20 ppm *m/z* window and eluting at approximately the same retention time (±0.2 min); in addition, the peak intensity of the group must be highly correlated (Pearson’s correlation coefficient *r* > 0.9) among all the samples, and the ratios of the detected peak intensities must satisfy the criteria: [M]/[M + 1] > 2 and [M + 1]/[M + 2] > 2. Software is available from https://github.com/AgResearch/peakOmics. Only the monoisotopic ion [M] of the series was retained for subsequent data analysis.

Batch effect was corrected using a parametric empirical Bayes methods (Johnson et al., 2007) using batch as a covariate. Additional filtering (*F*-test, *p*-values < 0.05) was further used to remove peaks still with the significant batch effect.

Among the replicates (*n* = 5), the most varied replicated groups were identified (cv > 0.2). The outliers were removed within the replicated groups if the modified *Z*-scores were >3.0, where modified *Z*-score is defined as *Z*_i_ = 0.6745^∗^[x_i_ – median(x)]/mad(x) and where mad is the median of absolute deviation (Iglewicz and Hoaglin, 1993). Invariant peaks among the ryegrass populations (Kruskal test, *p*-value > 0.05) were also removed.

Various statistical procedures such as PCA, clustering analysis, ranking algorithms were employed to extract information in each data-mining step in Results.

### Metabolite Annotation

After two de-isotoping procedures (CAMERA method, and our in-house method) a peak annotation table (including detected *m/z*, rt, *m/z* range, rt range, adduct, isotopic peaks of each peak) was created for annotation purposes throughout the study. Different annotation strategies were used for different classes of compounds. For flavonoid glycosides, in-source fragmentation patterns usually provide sufficient information for the annotation (von Roepenack-Lahaye et al., 2004; Abrankó et al., 2011; Pinheiro and Justino, 2012). In this study, annotation is performed on the aglycone types and the type and number of sugar moieties, but not on the glycosylation position nor inter-glycosidic linkages in glycan moieties. For other small metabolites such as plant alkaloids, we exploit data on the accurate mass, expected retention time, formula prediction and matched isotopic patterns for the annotation as detailed previously (Cao et al., 2015) along with published results. The spectral data supporting all the annotated metabolites mentioned in this publication were provided as a NIST (National Institute of Standards and Technology) MSP library and are available from https://github.com/AgResearch/peakOmics.

## Author Contributions

MC conceived the scope of the paper, conducted data analysis and implemented computer code to support data analysis and peak annotation. KF oversaw LCMS lab operations. MC and KF carried out peak annotation. CJ undertook funding acquisition, project coordination and management. AS provided plant resources. All authors conducted data interpretation and contributed to writing the paper.

## Conflict of Interest Statement

The authors declare that the research was conducted in the absence of any commercial or financial relationships that could be construed as a potential conflict of interest.
