# Supplementary material for: Untargeted Metabotyping Lolium perenne Reveals Population-Level Variation in Plant Flavonoids and Alkaloids
Source: Front Plant Sci. 2017 Feb 7;8:133. doi: 10.3389/fpls.2017.00133 (PMC5293862; doi:10.3389/fpls.2017.00133)
Supplement: Supplementary file 1 [file Data_Sheet_1.DOCX]

Supplementary Material

**Untargeted Metabotyping *Lolium perenne* Reveals Population-Level Variation in Plant Flavonoids and Alkaloids**

Mingshu Cao^1*^, Karl Fraser^1^, Chris Jones^1, 2^, Alan Stewart^3^, Thomas Lyons^1, 4^, Marty Faville^1^, Brent Barrett^1^

^1^AgResearch Grasslands Research Centre, Palmerston North 4442, New Zealand

^2^Present address: International Livestock Research Institute, PO Box 5689, Addis Ababa, Ethiopia

^3^PGG Wrightson Seeds, PO Box 175, Lincoln, Christchurch 7640, New Zealand

^4^Present address: PGG Wrightson Seeds, Palmerston North 4442, New Zealand

*** Correspondence:** Mingshu Cao: [mingshu.cao@agresearch.co.nz](mailto:mingshu.cao@agresearch.co.nz)

# Supplementary Data

## Supplementary Data 1 The list of top 50 CP peaks among the 23 populations; and a clustering of the 50 peaks based on the correlation of peak intensity.

[1] "CP127.0389_5.88" "CP187.06_5.45" "CP187.06_5.09" "CP521.1284_5.46" "CP109.0284_5.89"

[6] "CP317.1486_5.91" "CP187.0599_5.91" "CP85.0285_5.46" "CP653.1703_5.11" "CP317.0653_5.45"

[11] "CP127.0389_5.46" "CP317.1124_5.87" "CP127.0154_5.93" "CP317.0652_5.94" "CP317.0652_5.34"

[16] "CP303.0497_5.35" "CP303.0496_4.62" "CP304.0838_4.57" "CP303.0497_5.66" "CP521.1286_5.89"

[21] "CP303.0497_5.46" "CP522.3056_3.76" "CP288.159_5.17" "CP317.0653_4.89" "CP667.1858_5.46"

[26] "CP507.1128_5.59" "CP303.0496_5.18" "CP127.0389_5.31" "CP85.0285_4.59" "CP109.0284_5.17"

[31] "CP303.0496_4.99" "CP85.0285_5.31" "CP127.0389_5.17" "CP465.1024_4.98" "CP231.0495_4.15"

[36] "CP697.1593_4.15" "CP145.0494_5.17" "CP434.2167_4.8" "CP449.1071_4.16" "CP535.1073_4.15"

[41] "CP159.0285_4.15" "CP287.0544_4.15" "CP287.0548_5.84" "CP163.0386_3.86" "CP449.1071_4.04"

[46] "CP135.0439_3.86" "CP287.0545_4.04" "CP158.0599_3.86" "CP89.0386_3.86" "CP337.0912_3.86"

**Supplementary Data 2** The list of top 50 CN peaks among 23 the populations; and a clustering of the 50 peaks based on the correlation of peak intensity.

[1] "CN651.1567_5.07" "CN665.2085_5.39" "CN505.0985_5.54" "CN300.0272_5.55"

[5] "CN651.1564_5.29" "CN609.1458_4.59" "CN623.1616_4.83" "CN519.1141_5.85"

[9] "CN245.0121_4.18" "CN96.9595_4.19" "CN477.0945_5.31" "CN635.1615_5.37"

[13] "CN595.1307_4.56" "CN96.9595_3.06" "CN463.088_4.95" "CN300.0272_4.95"

[17] "CN695.133_4.13" "CN651.1563_4.13" "CN489.0942_4.13" "CN96.9595_4.28"

[21] "CN119.0497_5.83" "CN653.1501_4.13" "CN531.104_4.13" "CN695.1468_4.24"

[25] "CN353.0873_3.84" "CN191.0555_3.84" "CN489.1036_5.79" "CN478.1991_4.53"

[29] "CN489.1034_4.01" "CN651.1563_4.01" "CN707.1826_3.84" "CN207.0658_5.82"

[33] "CN111.0082_5.92" "CN610.138_3.92" "CN609.1344_3.78" "CN695.1332_4.03"

[37] "CN204.9889_5.98" "CN163.0395_5.9" "CN143.0344_5.9" "CN169.0137_5.92"

[41] "CN609.146_3.9" "CN351.0718_5.92" "CN205.0349_5.95" "CN351.129_3.9"

[45] "CN162.9819_6.01" "CN161.0814_4.66" "CN474.2623_9.04" "CN351.0651_3.9"

**Supplementary Data 3** The list of top 50 CP peaks among the 21 populations; and a clustering of the 50 peaks based on the correlation of peak intensity.

[1] "CP434.2167_4.8" "CP535.1075_4.04" "CP231.0496_4.04" "CP287.0545_4.04"

[5] "CP449.1071_4.04" "CP697.1598_4.04" "CP788.3112_4.03" "CP288.159_4.8"

[9] "CP434.2167_4.58" "CP287.1482_4.09" "CP163.0388_4.61" "CP287.0546_3.78"

[13] "CP287.1377_4.06" "CP158.0599_3.86" "CP287.1379_3.78" "CP118.0862_4.58"

[17] "CP337.0912_3.86" "CP163.0386_3.86" "CP211.1691_5.82" "CP186.0547_3.86"

[21] "CP135.0439_3.86" "CP89.0386_3.86" "CP130.065_3.86" "CP181.0492_3.86"

[25] "CP107.0491_3.86" "CP435.164_4.54" "CP117.0334_3.86" "CP187.126_3.87"

[29] "CP118.0651_4.55" "CP163.0388_5.14" "CP145.0282_3.86" "CP449.1072_3.77"

[33] "CP163.0388_5.53" "CP209.1533_4.04" "CP83.0492_4.04" "CP163.0387_4.23"

[37] "CP167.1428_4.04" "CP287.1381_4.48" "CP89.0386_4.23" "CP163.0388_4.45"

[41] "CP287.0547_4.56" "CP787.2278_3.96" "CP135.0439_4.23" "CP288.1591_4.58"

[45] "CP163.0388_4.09" "CP859.1917_5.21" "CP783.1605_4.57" "CP261.2209_8.94"

[49] "CP79.0543_3.86" "CP659.3629_8.94"

**Supplementary Data 4** The list of top 50 CN peaks among the 21 populations; and a clustering of the 50 peaks based on the correlation of peak intensity.

[1] "CN433.2076_4.02" "CN478.1991_4.53" "CN489.1034_4.01" "CN335.0769_4.42"

[5] "CN609.1344_3.78" "CN651.1563_4.01" "CN353.0875_5.39" "CN695.1468_4.24"

[9] "CN431.192_4.03" "CN695.1332_4.03" "CN625.1407_3.66" "CN639.1567_3.94"

[13] "CN353.0873_3.84" "CN191.0555_3.84" "CN191.0556_4.06" "CN353.0874_4.06"

[17] "CN707.1826_3.84" "CN351.0651_3.9" "CN179.0344_4.58" "CN191.0555_4.2"

[21] "CN610.138_3.92" "CN696.1369_4.03" "CN351.129_3.9" "CN367.1029_3.96"

[25] "CN191.0556_3.09" "CN521.3327_5.58" "CN354.084_4.2" "CN179.0556_4.41"

[29] "CN283.08_4.44" "CN335.1343_4.56" "CN368.0994_3.96" "CN96.9595_4.42"

[33] "CN179.0344_3.83" "CN341.0876_4.41" "CN675.3598_8.99" "CN675.3598_8.79"

[37] "CN191.1106_5.35" "CN609.146_3.9" "CN179.0344_3.92" "CN353.0873_4.2"

[41] "CN593.1507_5.02" "CN721.3656_8.99" "CN193.0501_3.96" "CN721.3654_8.79"

[45] "CN435.0598_4.04" "CN161.0814_4.66" "CN191.0556_5.39" "CN397.1274_8.79"

[49] "CN161.0238_3.84" "CN191.0556_4.98"

**Supplementary Data 5** Annotation data for CP788.3112_4.03. (A) This peak is significantly varied among the 23 populations (Empirical Bayes, FDR p-value < 0.01), which is the highest in Samson, but with at the noise-level in PG238, Tunisia, Barlet, FLp0971 and Tolosa. (B) there are 2 closely eluting peaks. The rt range of this detected peak is [238.4, 248.7], therefore the mass spectrum from the first peak was calculated as shown in (C). This peak can be annotated as K-Glc-feruloylGlc. The loss of *m/z* 339.20 in the mass spectra indicates the presence of feruloylGlc in the molecule.


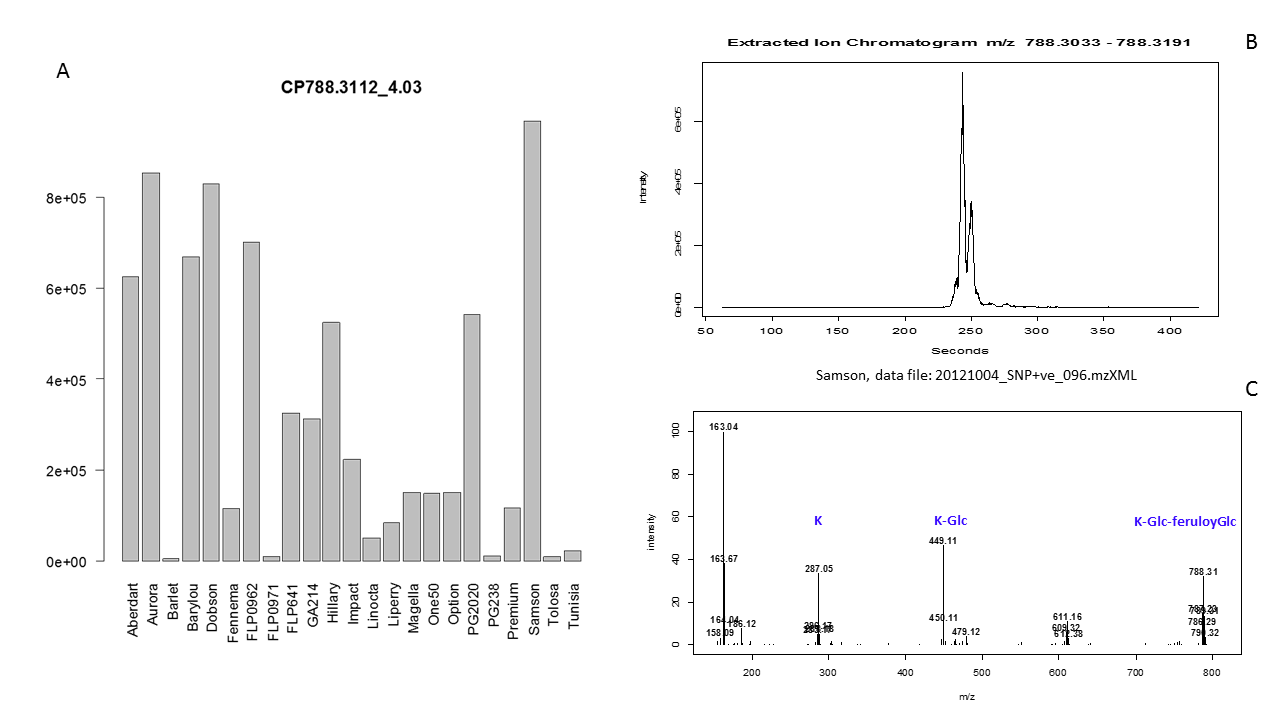


**Supplementary Data 6** Validation data for the two annotated peaks.

Plants used in this study were harvested in May 2012 (late autumn). These plants have been continuously maintained in pots (outdoor) and in the same location, Palmerston North, New Zealand. We carried out another harvest in September 2013 (early spring) for the validation purpose. This new harvest followed the same sample preparation and the same analytical procedures as described in METHODS. Samples for this harvest were stored at -20ºC and underwent C18 LCMS assays from March 2015.

We selected two peaks, i.e CP697.1593_4.15 (annotated as K-Glc-maGlc) and CN353.0875_5.39 (mixture, but predominantly represented for chlorogenic acid glycoside), which were reported as differentially expressed among populations. CP697.1593_4.15 (K-Glc-maGlc) was absent in both population ‘PG238’ and ‘Tunisia’; and CN353.0875_5.39, a relatively weak peak but consistently present in ‘PG2020’ genotypes. Therefore, from the validation data set we selected 4 data files generated from genotypes of ‘Tunisia’, ‘PG2020’ and ‘Impact’ (see data description below). These data in mzXML format can be also used for the validation of other peaks, and are available from <https://github.com/AgResearch/peakOmics>

*Description of the validation data*

**data file*** **ryegrass** **population** **ion mode**

-----------------------------------------------------------------------------------------------------------------

04052015_PG3_Batch7_SNP+ve_026.mzXML Impact positive

11052015_PG3_Batch6_SNP+ve_018.mzXML Tunisia positive

04052015_PG3_Batch7_SNP-ve_026.mzXML Impact negative

23032015_PG3_Batch1_SNP-ve_066.mzXML PG2020 negative

------------------------------------------------------------------------------------------------------------------

*the date of running sample is encoded in the file name.

*Peak validation of CP697.1593_4.15*

As shown in the EIC of 697.1593 (±10 ppm) from two sample selected from Tunisia and Impact population, this proves that the metabolite K-Glc-maGlc represented by *m/z* 697.1593 [M+H]^+^ is absent in Tunisia. We have investigated the mass spectrum eluted around the two major peaks shown in EIC, the mass spectrum derived are similar with the same set of ions and only slight variation on the relative intensity of the ions, indicating our annotation is valid at the level we followed in our study, the subtle structural variation of K-Glc-maGlc isomers cannot be approached due to the lack of further evidence. Also shown is the mass spectrum eluted from the most intense peak (253 ± 1 sec, 4.22 min).

*The peak validation of CN353.0875_5.39*

As shown the peak CN353.0875_5.39 is also reproducible in the two years. This peak is consistently present in ‘PG2020’ population. The mass spectrum derived from 329 sec (± 1 sec, 5.48 min) shows that it is a mixture of chlorogenic acid glycoside (353.09, 515.12 m/z) and a flavonoid derivative.

# Supplementary Figures and Tables

## Supplementary Figures

**Supplementary Figure 1.** PCA analysis bases on the raw CP peaks before filtering. The plot 36 batches are color-coded. The score plot shows the predominant batch effects with samples from different genotypes and populations form its own batch cluster. The same score plot is shown with batch labels as outliers from batch 15 and 16 can be seen.

**Supplementary Figure 2.** PCA analysis based on 15,486 CN peaks generated from 1269 samples, with PC1 accounts for 4.3% of total variation and PC2 for 3.9%. Besides of Controls (n=80), PG238 samples are also differentiated from other populations.

**Supplementary Figure 3.** LDA analysis among 21 populations (without ‘PG238’ and ‘Tunisia’). Data were based on the top 500 CP peaks.

**Supplementary Figure 4.** Peak CN353.0875_5.39 is only present in the PG2020 population, as shown EIC data of 6 samples from PG2020 and 6 samples from Impact.

**Supplementary Figure 5.** Perloline variation among the 23 ryegrass populations.

## Supplementary Tables

**Supplementary Table 1.** Overview of the samples used in this study. Columns represent ryegrass population names, the number of genotypes (individuals) from each population, the number of samples run in positive ion mode and the number of samples run in negative ion mode.

| **population** | **noGeno** | **noSample_pos** | **noSample_neg** |
| --- | --- | --- | --- |
| Aberdart | 12 | 60 | 62 |
| Aurora | 5 | 25 | 28 |
| Barlet | 7 | 35 | 34 |
| Barylou | 6 | 30 | 29 |
| Dobson | 6 | 30 | 31 |
| Fennema | 7 | 34 | 33 |
| FLp0962 | 22 | 110 | 111 |
| FLp0971 | 11 | 55 | 57 |
| FLp641 | 25 | 124 | 123 |
| GA214 | 23 | 116 | 120 |
| Hillary | 3 | 15 | 17 |
| Impact | 4 | 20 | 22 |
| Linocta | 5 | 25 | 26 |
| Liperry | 4 | 20 | 22 |
| Magella | 6 | 30 | 26 |
| One50 | 4 | 20 | 18 |
| Option | 11 | 54 | 59 |
| PG2020 | 23 | 114 | 114 |
| PG238 | 13 | 61 | 68 |
| Premium | 11 | 55 | 57 |
| Samson | 4 | 20 | 23 |
| Tolosa | 13 | 65 | 70 |
| Tunisia | 8 | 39 | 39 |
